# Gaze dynamics of cued speech perception

**DOI:** 10.1038/s41598-026-40719-9

**Published:** 2026-06-12

**Authors:** Annahita Sarré, Laurent Cohen

**Affiliations:** 1 Inserm U 1127, CNRS UMR 7225, Sorbonne Universités, Institut du Cerveau, ICM, Hôpital Pitié, 47 Boulevard de l’Hôpital, Paris, 75013 France; 2 AP-HP, Hôpital de La Pitié Salpêtrière, Fédération de Neurologie, Paris, 75013 France

**Keywords:** Deafness, Language, Eye-tracking, Cued speech, Face perception, Human behaviour, Language, Perception

## Abstract

For many deaf people, lip-reading plays a major role in verbal communication. However, lip reading is inherently ambiguous and does not allow for a complete understanding of speech. The consequences of these limitations are significant, potentially impeding language, cognitive, and social development. Cued speech (CS) was developed to eliminate this ambiguity by supplementing lip-reading with hand gestures, giving access to the entire phonological content of speech through the visual modality alone. Despite its documented efficacy in enhancing linguistic and communicative abilities, the mechanisms of CS perception remain largely unknown. The present study is the first to examine eye movements during CS perception, with a sample of deaf CS users, hearing CS users, and hearing naive controls. We presented silent videos of words, pseudowords, and sentences in their CS form, while recording the participants’ eye movements. All groups fixated almost exclusively on the face, and predominantly on the lips of the speaker, despite the effective processing of CS gestures by CS users. Deaf and hearing participants differed strikingly in the way fixation was distributed between the left and right halves of the face. While both hearing groups mostly fixated the left side of the speaker’s face, deaf participants showed a more symmetrical pattern. Finally, in CS users, stimuli that were phonologically, lexically or semantically more difficult tended to increase fixation in the inferior and left sector of the face. Apart from reading, CS stands as the sole system for visually conveying full phonological information of a spoken language. This study elucidates the fundamental behavioral tuning that facilitates the efficient recovery of phonology in this distinctive form.

**Supplementary Information:**

The online version contains supplementary material available at 10.1038/s41598-026-40719-9.

## Introduction

Audition is the primary channel for accessing spoken linguistic information, enabling speech perception and verbal communication. However, in deaf individuals, vision assumes a disproportionate role, compensating for the absence of auditory input and becoming central to language processing through modalities such as sign language and speechreading. Profoundly deaf individuals typically rely on one of two main options for language acquisition: sign languages, which are fully autonomous visual languages accessible to deaf persons with no mediation of spoken language, or spoken languages supported by lip-reading and often supplemented with speech therapy, hearing devices and assistive systems such as cued speech (CS).

For these two approaches, the efficient capture of visual information relies on precise control of eye movements, which directs attention to relevant linguistic cues and optimize information intake. Studying eye movements in deaf and hearing individuals provides crucial insights into the neural and cognitive adaptations supporting visual language processing. Despite the existence of cued speech as a system for visually conveying the phonological content of speech, few studies have investigated its mechanisms^1–10^, and no study has yet examined eye movements during cued speech perception. The present study addresses this gap, offering novel insights into the visual strategies supporting cued speech processing.

Cued speech (CS) was designed by Dr. R. Orin Cornett^11^ to counteract the ambiguity of lip-reading using hand gestures to give access to the entire phonological content of speech through the visual modality. The original American CS system was adapted to over 65 languages and dialects (International Academy Supporting Adaptations of Cued Speech^12^, including French where the system is referred to as “Langue française Parlée Complétée” (LfPC). CS is typically used in complement with cochlear implants or, less often, hearing aids, and it significantly improves the general linguistic skills of deaf users^13,14^.

CS speakers decompose words into consonant-vowel (CV) syllables (e.g. “pari” → /pa-ʁi/), sometimes requiring adjustments (e.g. “drakkar” → /d-ʁa-ka-ʁ/ instead of /dʁa-kaʁ/). The identity of each syllable is then conveyed through 3 cues: the lip movements, the position and the shape of the hand. The hand assumes one among 8 possible shapes (e.g. index extended and other fingers folded), each shape representing ~ 3 consonant phonemes (e.g. /p/, /d/ and /ʒ/). In parallel, the hand is placed in one among 5 possible positions relative to the face (e.g. next to the chin), each position representing ~ 3 vowel phonemes (e.g. /a/, /o/, and /œ/). The system is designed such that two syllables sharing the same lip movements will be supplemented by two different hand gestures, allowing for an easy differentiation. To a given combination of the three clues thus corresponds a unique syllable, allowing CS to convey phonology through the visual modality.

Research on eye movements in language perception has primarily focused on lip-reading and sign language perception, plus the ancillary fingerspelling system, which are all relevant to profoundly deaf individuals. In both cases, the pattern of fixation tends to conciliate the optimization of verbal information intake with the adaptation to social cues and norms, as we will now review.

During lip-reading, the mouth is fixated more than the eyes, all the more so that comprehension is difficult, due to task demands^15–17^, to the use of an unfamiliar language^18–20^, or to the presence of noise^17,21,22^. Note however that looking directly at the mouth may not always be necessary for sufficient visual information intake and that peripheral vision may at times suffice, as evidenced by Yi et al.^23^, and by the studies finding mixed results on the influence of direct mouth fixation on occurrence of the McGurk-MacDonald effect^22,24,25^. Moreover, several studies trigger audiovisual integration with low quality visual facial cues – induced by distance of the speaker or artificially reduced spatial frequency –, so that foveal spatial resolution may not be crucial for this processing^26–28^.

Conversely, social influences tend to attract people’s gaze to their interlocutor’s eyes, which act as a strong exogen attractor (see Frischen et al.^29^ for a review), an effect that is subtly modulated by cultural norms and social context^30–37^.

Finally, while gestures contribute important information during speech perception^38–40^, all eye-tracking studies conclude that the gaze remains oriented towards the face, while gestures themselves are almost never fixated^41–44^,but see^45^, except when speakers suddenly hold their gesture^44,46,47^, or look at their own hand^44,46^. Moreover, we found no convincing evidence that gesture location may affect gaze direction^46,48^. In fact, two studies report a discrepancy between direct gaze at gestures and their uptake^42,46^, but see^49^. In summary, peripheral vision seems to be sufficient for gesture processing.

During the perception of signed languages, which largely rely on manual articulators, it is remarkable that all eye-tracking studies found that fixation is primarily oriented towards the signer’s face^50–54^. Similar to lip-reading, it is mostly during occasional moments of joint attention that the two interlocutors fixate the hands, particularly during the production of “classifier” signs^50,52^; see examples of classifiers in Cogill-Koez^55^.

Importantly, face movements also play an important role in the prosody, syntax or morphology of a signed language (examples in ASL^56)^. Moreover, the “signing space” often extends from the lower torso to the upper part of the face, and the location and subtility of the information are mostly unpredictable. Hence, fixating the signer’s face appears as a fruitful strategy, as it allows both to grasp the most subtle facial signals, which would be hard to perceive in the periphery, and to process the more salient hand movements even away from the fovea, without needing to predict their location. Interestingly, Schotter et al. (2020)^57^ found that meaningful ASL signs recognition was less affected by eccentricity of space of presentation compared to pseudosigns. This strategy may thus come at its limit when the word conveyed is entirely unpredictable, inciting signers to overtly gaze at the hand. Accordingly, in Gappmayr & Lieberman^54^ pseudosigns triggered more overt fixations to the hand than real signs, and fingerspelled pseudowords more than fingerspelled real words.

Evidence for social modulation of gaze behavior remains scarce in the literature of signed languages gaze behavior. According to Emmorey et al.^50^, native signers tend to look at the signer’s eyes, so that they would focus on social aspects as seen with spoken language addressees.

Finally, when communicating through a sign language, an ancillary fingerspelling system may be used to convey the spelling of words, for instance proper nouns^58^. Similar to lip-reading and signed languages, fingerspelling did not cause the gaze to shift away from the signer’s face, either with the bimanual British Sign Language (BSL) fingerspelling^51^, or with the unimanual ASL system^50^. Fixation of the signing hand occurred only with unnatural stimuli featuring no facial movements^54^. Considering the overall visual similarity of fingerspelling and CS gestures – both consist of handshape and are used to convey the phonology of a given spoken language through the visual modality –, one may predict that a similar gaze behavior should prevail with CS.

In summary, gaze behavior during visual language perception reflects a trade-off between the intake of information, which is influenced by the predictability and the complexity of the input, and the reaction to and compliance with social cues and norms. The critical information in those two domains roughly predominates in the regions of the mouth and of the eyes, respectively. Furthermore, regardless of their meaning and perceptual features, hand gestures are generally processed through peripheral vision and not overtly looked at.

In the current study of gaze fixation during cued speech perception, we will assess the following four predictions and questions.

### Gaze distribution between the hand and the face

In audiovisual speech, signed language, and even fingerspelling, the gaze stays almost exclusively directed towards the face and away from informative hand gestures, and hence one may safely predict that such will be the case also for CS. Further supporting the prediction that movements to the periphery would be superfluous, CS gestures are produced very close to the face, and CS perceivers are excellent at understanding lip-reading even in the absence of CS gestures^59,60^, so that they may continue to focus on lips even when CS is available. However, one could argue contrariwise that those two factors could make it easier and faster for CS users to glance at the gestures. Moreover, CS gestures are produced earlier than the lip movements^4,5,10^, which could allow the perceivers to rapidly anticipate the syllable based on the current gestures. Finally, deaf users of CS attribute more weight to the hands than to the lips whenever they provide incongruent cues^2,6,8^. Overall such mechanisms may push CS users to move their eyes to the periphery, despite their likely preference for the face area.

### Gaze distribution between the eyes and the mouth

Our second prediction is that fixation to the face should be predominantly directed towards the mouth, rather than the upper part of the face, like during ordinary lip-reading. Indeed, the same phonological information should be acquired through lip-reading during the perception of audiovisual speech and of purely visual CS. Moreover one may speculate that hearing users of CS, who are often not excellent lip-readers, will fixate the mouth more than the skilled deaf users, in order to maximize phonological information intake.

### Gaze distribution between the left and the right sides of the face

The contribution of the left and right visual hemifields to visual speech perception is subject to potentially conflicting influences. On the one hand, visual speech is better decoded whenever the whole target face is presented in the right hemifield^61,62^, an advantage possibly related to the usual left hemispheric dominance for language. Note that an opposite left hemifield advantage was reproducibly observed during judgments of non-verbal features such as identity, emotion or gender (review^63^). On the other hand, the right side of the speaker’s mouth is more mobile during speech production^64^ and more informative for language comprehension^65^. Whenever the speaking face falls at the center of the visual field, this bias yields an advantage of the *left* hemifield for information intake. The latter asymmetry may explain why viewers fixate preferentially the left half of speaking faces^66,67^. In CS, such leftward fixation bias could be reinforced by the presence of informative hand gestures, which typically appear in the left hemifield. We would therefore predict a general leftward bias during CS perception. However, studies assessing the gaze dynamics of deaf individuals during reading have shown a reduction of the usual right visual field advantage when processing written words^68,69^, and an expanded visual reading span to the left of fixation^70^. There is also enhanced processing of visual stimuli in the peripheral visual field of deaf compared to hearing people, although the literature is not fully consistent on this matter, possibly due to the heterogeneity of the deaf population^71,72^. This phenomenon could contribute to erasing the usual leftward fixation bias in deaf CS users. In this study, we will thus determine whether deafness and mastery of CS will alter this default pattern.

### The impact of stimulus difficulty

To assess our predictions, we tracked the gaze of three groups of participants – deaf CS users, hearing CS users and hearing naïve controls – during the perception of silent videos of a person producing words, pseudowords or sentences in CS. We studied how fixation is distributed between the hand and the face, the lips and the eyes, the left and the right.

We also manipulated the intrinsic difficulty of stimuli, by contrasting (i) real and pseudo words, (ii) high- and low-frequency words^73^, (iii) words whose number of CS syllables matches or not the number of spoken syllables, as mismatch causes word identification errors^1^, and (iv) words that are predictable or not based on sentential context.

## Methods

### Participants

We recruited 63 volunteers: 22 prelingually and severely/profoundly deaf CS users, 21 hearing CS users and 20 hearing controls with no knowledge of CS. Participants were 18–65 years old French native speakers. All participants were right-handers according to the Edinburgh inventory^74^ except for 1 deaf and 2 hearing ambidextrous CS users. They had no history of neurological or psychiatric disorders, and all had normal or corrected to normal vision. They did not wear contact lenses or eye make-up during the acquisition.

Because this study focuses on a rare population, our goal was to recruit as many participants as possible who met the requirements. Around 20 people was the maximum number that could be achieved after acquiring data for several years, and extending the recruitment to all of France and to neighboring French-speaking countries. The statistical power of the present study can thus be considered as high as it can be.

For the two groups of CS users, CS comprehension and production were assessed before the experimental session. Importantly, deaf participants were required to have a fluent level in CS production and comprehension, and hearing participants to master CS production but not necessarily expert comprehension. Indeed hearing users typically master CS production better than perception, as they use CS to address deaf relatives or people whom they assist, but are rarely addressed to in silent CS. They also generally learn CS as adults. We did not recruit deaf non-CS users, as prior work suggests broadly similar gaze behavior in spoken and signed language, making it unlikely that sign language proficiency would meaningfully affect CS gaze patterns. Recruitment of CS users was done through social networks and mailing lists of CS associations.

Before the experimental session, all participants were asked to fill a questionnaire with general demographic questions. Deaf CS users were asked additional questions on the etiology and severity of deafness, on the history of language acquisition, on hearing aids and on the daily use of CS. Hearing CS users only answered the questions on the learning of CS and its use in daily life.

Deaf participants self-reported being severely (*n* = 1), profoundly (*n* = 19) or totally (*n* = 2) deaf. All were pre-linguistically deaf, including 20 out of 22 cases of congenital deafness. They reported standard ages of reading acquisition (5.23 ± 1.38 years old), with good to excellent current reading capacities. All deaf users had hearing devices: 14 deaf participants possessed bilateral hearing aids (time of daily use: 11.43 ± 4.94 h), 4 had two cochlear implants, and 3 had one cochlear implant (age of implementation: 14.86 ± 9.97 yo; time of daily use: 8.43 ± 4.93 h). All external parts of the devices were removed before the experiment to permit the installation of an EEG cap.

Hearing users and controls had normal hearing, except for one Hearing CS user who had moderate deafness.

The three groups were matched in age (Deaf: 35.2 ± 8.6 yo; hearing: 35.7 ± 10.2 yo; controls: 34.9 ± 10.9 yo) and education level (university degree: deaf: *n* = 20; hearing: *n* = 19; controls: *n* = 18). The sex ratio did not differ between the Deaf and Controls groups (Deaf: 15 F; controls: 13 F), while the hearing users of CS included slightly more females (18 F). Deaf and hearing CS users differed in their age of CS learning (Deaf: 2.7 ± 2.6 yo; hearing: 22.2 ± 10.5 yo). 4 CS users used CS less than once a month, and were proficient deaf early CS users who scored high on the pretest. All the others used CS at least once a month, and a large majority daily or several times a week (Deaf: *n* = 14; hearing: *n* = 18). Moreover, 17 Deaf and 13 Hearing users declared having some knowledge of the French Sign Language, with a later age of learning than for CS for deaf participants (Deaf: 16.8 ± 12.3 yo; Hearing: 21.8 ± 9.9 yo). These participants had varying self-declared levels in comprehension and production, with at least an occasional and regular use by Deaf participants (12 using French Sign Language at least once a month).

All information was provided in written form identically to the 3 groups of participants, who all gave their informed consent. The experiment was approved by the Comité de Protection des Personnes ILE DE FRANCE X (N° CPP 2022-A00847-36) and carried out in accordance with the Declaration of Helsinki.

### Stimuli and experimental design

Two distinct experiments were carried out, each based on the presentation of silent videos of a person producing CS, either lists of real words and pseudowords (Experiment I) or sentences (Experiment II). A different professional CS coder produced the material of each experiment. Stimuli used the French version of CS, the “Langue française Parlée Complétée” (LfPC). For CS users, these two experiments were preceded by a short behavioral assessment of CS proficiency.

#### Behavioral assessment of cued speech comprehension

The proficiency of CS users in CS comprehension and production was assessed before the eye-tracking session. Comprehension was tested through a short dictation test, where the participants had to write down 12 CS sentences presented as silent videos. Each sentence was presented twice, and participants had to respond after each viewing. CS production was tested by asking participants to transpose 12 written sentences into CS. The accuracy and fluency of responses were checked by the experimenter. For CS comprehension, responses were assessed by computing for each sentence the percentage of correctly transcribed phonemes. Spelling mistakes were disregarded as long as they transcribed the correct phoneme. Results were then averaged across sentences.

For both tests, sentences were distributed into three levels of difficulty. “Easy” sentences were short, used the present tense, included only frequent and semantically predictable words, and CV syllables. “Intermediate” sentences were short, used various tenses, and included frequent but less predictable words and one complex syllabic pattern (V-CCV, V-CVC or VC-CV)^1^. “Difficult” sentences were longer, used various tenses, included less frequent and less predictable words, and one complex syllabic pattern.

#### Experiment I – words

Words were selected from the MEGALEX database^75^, following a design crossing frequency (high or low) x mismatch (level 0, 1 or 2), and differed in terms of frequency (high or low), of Syllabic-CS gestures mismatch (level 0, 1 or 2) and of lexicality (real or pseudo word). This resulted in a fully crossed design.

Word frequency was estimated as the logarithm of the sum of the frequencies in books and films corpora in the MEGALEX database. Frequent words had a frequency > 1, and infrequent words a frequency < 0.5.

With the mismatch factor, we manipulated the difference between the number of spoken syllables and the number of CS gestures. Words all required 3 CS gestures, while their number of spoken syllables ranged from 3 to 1, which implies a mismatch of 0 to 2. For example, the French word “poison” has a mismatch level of 1, because its production requires 3 CS gestures: /p-wa-zɔ̃/, while it contains 2 syllables: /pwa-zɔ̃/). We used an equal number of stimuli for mismatch levels 0, 1, and 2.

40 words were selected in each frequency x mismatch category. We found no interaction between frequency and mismatch (*p* = 0.45), so that the two factors were orthogonal.

Finally, we manually derived real words present in the MEGALEX database to create 30 phonologically legal pseudowords. While frequency is irrelevant in the case of pseudowords, these stimuli were evenly distributed within the 3 Syllable-CS gestures congruency values. Pseudowords represented 1/8 of stimuli.

Deaf and hearing CS users were presented with the resulting 240 words and 30 pseudowords. They were instructed to press the mouse button whenever they detected a pseudoword. Stimuli were presented in a random order, distributed into 2 runs of 9 min. All categories of stimuli were equally distributed between the two runs.

As Control participants had no understanding of CS, they received a shorter version of the experiment, with 10 randomly selected words (*N* = 60) per category plus 12 pseudowords, for a total duration of 5 min. They were instructed to remain attentive and “to understand what they could understand”.

#### Experiment II – sentences

Pairs of sentences ending with the same word were created. One sentence predicted the final word with a high probability (“I hear the meowing of a CAT”), while the other sentence did not allow to predict the word, whose occurrence was nevertheless plausible (“I’m going to buy a CAT”). Sentences from the same pair shared a similar grammatical structure, the same number of words (+/- 1), and the same exact number of CS gestures. Some sentences were adapted from the material from Peelle et al. (2020)^76^ and Robichon et al. (1996)^77^. The predictabilities of the final words w assessed by asking native French speakers between the ages of 18 and 65 to write the first word that came to their mind in order to complete the sentences, presented in written form with the last word lacking. We selected pairs of sentences for which (i) the predictive sentence was completed with the target word in over 60% of responses, and (ii) the non-predictive sentence was completed with the target word in less than 15% of the responses, plus any variety of words. This resulted in the selection of 50 sentence pairs.

Deaf and hearing CS users viewed each sentence once, distributed in 2 runs, such that (i) the two sentences of a pair were always displayed in different runs, and (ii) each run contained an equal number of predictive and non-predictive sentences.

Participants were asked to try their best to understand the sentences, and were regularly tested for their comprehension to support their motivation. A written sentence was presented once every 12–13 videos, and participants were asked to indicate whether it had appeared before among the CS stimuli. Half of these test sentences had been in CS among the 12–13 previous videos, and half were taken from a distinct list. A total of 8 such test trials were presented.

As in Experiment I, Control participants were presented with a subset of the stimuli and instructed to try their best to understand the sentences. The 15 pairs of sentences with the larger predictability effect in the pilot online questionnaire were presented, in random order, during a single 5 min run.

### Stimuli filming and edition

Stimuli were recorded with two professional coders. Videos were edited using the Movavi software and the moviepy Python module. They were silenced and trimmed so that each video started when the hand appeared in the frame for the first gesture (that is at least 500ms before speech onset), and ended 400ms after the end of the speech output. A 200ms fade in/out effect to a fixation cross started and ended each video. This grey cross was presented on a uniform background similar to the videos’, and was displayed during 0.3–0.9 s. The interstimulus interval was of 0.8 s. The mean length of resulting videos was of 7.79 ± 1.34s for sentences and of 2.2 ± 0.399s for words.

### Apparatus and data recording

Eye movements and pupil area were recorded using the 35 mm monobinocular lens of the SR Research EyeLink 1000 Plus version 1.2 apparatus for stabilized head, at a sampling rate of 1 kHz.

Stimuli were displayed to the participants on an ASUS VG32AQL1A monitor with a resolution of 2560/1440 pixels for a 700/395 mm screen. Triggers from the stimulus computer (OS Windows 10) were sent to the acquisition system using a parallel port.

The scripts for all stimulation programs were written in MATLAB using Psychtoolbox Version 3^78^.

### Procedure

Participants were seated in front of the screen with their head on a chin rest, with their eyes 700 mm away from the screen. The angular size of the head of the coder presented in the video corresponded to the angle perceived during actual conversations entertained at a normal social distance^79^.

Each participant’s dominant eye was recorded. The study started with the calibration and validation of the eye-tracker. The quality of eye-tracking acquisition was monitored along the study. Drift check and, if necessary, recalibration and validation were performed before each run. The EyeLink 5-points manual calibration was typically used. Exceptions came from a few participants for which calibration at the corners of the screen was impossible. In such cases, we switched to the 3-points calibration that does not include such points. As participants did not look at the corners when performing the experiments, this did not affect the quality of the data for these participants. For all calibrations and drift checks, we respected the guideline of SR Research of having less than a 0.5° drift.

Participants were encouraged to rest between runs if needed.

### Preprocessing

The first steps of preprocessing were performed online by the EyeLink apparatus, using the default parameters. Saccades, blinks and fixations were identified using the “normal” saccade sensitivity setting, corresponding to a 30°/sec velocity threshold, and the “extra file sample” filter for data smoothing.

After recording, two subjects were excluded from the analyses: One control was excluded from both experiments because they had not looked at the coder nor at the fixation cross, one control was excluded from the Words experiment for the same reason, and one deaf user was excluded from the Words experiment analyses because of a file loss.

We computed the proportion of track loss in each trial to further exclude bad trials and participants. Reasons for track loss were diverse, including blinking, prolonged eye closure, off-screen signal and loss of signal through eye-tracking for technical reasons. To avoid intergroup inconsistency, we only based participant exclusion on trials displayed to all participants.

In the two experiments, trials with a track loss of 25% and higher were discarded, leading to a global exclusion of 3.92% of Word trials and 2.49% of Sentence trials. This threshold of 25% was chosen for both experiments separately, as the best compromise between data quality and quantity. For each experiment, we then excluded participants for which more than 50% of trials were discarded: two deaf users for the Words experiment and one control for the Sentences experiment. These exclusions led to a final attrition rate of 3.11% of Word trials and 1.43% of Sentence trials. Considering each experiment separately, all participants had less than 25% of excluded trials and all stimuli had less than 7% rejected occurrences.

Following all the exclusions, the final study population was 19 deaf CS users, 20 hearing CS users and 18 hearing controls for the Words experiment, and 22 deaf CS users, 20 hearing CS users and 19 hearing controls for the Sentences experiment. The resulting repartition of trial conditions did not differ from the original design (Chi-square test: *p* > 0.05 for each condition), and these repartitions did not differ between the three groups (Chi-square test on a contingency table: for Words χ^2^(2) = 0.62, *p* > 0.05; for Sentences χ^2^(2) = 0.001, *p* > 0.05).

### Definition of AOIs

**Fig. 1:**
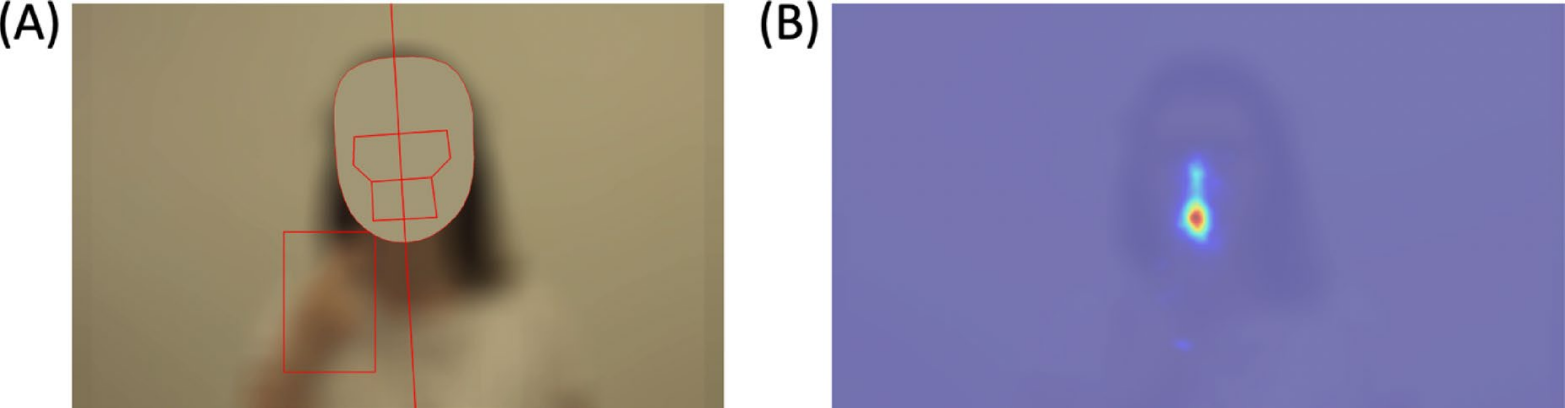
(**A**) Definition of the areas of interest for the lips, the eyes, the whole face, and the hand. The facial regions are divided in their left and right halves; (**B**) Heatmap of gaze orientation during the perception of cued speech words in all participants.

For each video, we extracted the coder’s body and face Mediapipe coordinates for each frame^80^ and used relevant coordinates to automatically draw each Area of Interest (AOI): the hand, the face, the eyes, the lips, the nose, and the vertical axis of facial symmetry used for the analysis of gaze asymmetry (Fig. 1A).

Most fixation data points fell in these AOIs, in the Words experiment (78.6% in deaf; 78.4% in hearing; 82.9% in controls) as in the Sentences experiment (90.7% in deaf; 91.2% in hearing; 89.9% in controls).

Because CS hand positions are located near the head, the hand AOI frequently overlapped with the face and lips AOIs. However very few fixations fell in these regions of overlap, in the Words (hand and face overlap: 1.6% in deaf; 4.2% in hearing; 2.2% in controls / hand and lips overlap: 1.2%in deaf; 3.0% in hearing; 1.7% in controls) as in the Sentences (hand and face overlap: 1.2% in deaf; 2.4% in hearing; 2.3% in controls / hand and lips overlap: 0.7% in deaf; 1.1% in hearing; 1.0% in controls) experiments.

### Analysis of eye movements

Analyses were performed using custom-made Python code, regularly using the MNE module^81^. Statistical tests used the pingouin and scipy modules for pretest behavioral analyses, and the jmv R package (imported in Python) for gaze analyses.

We performed binary comparisons of proportions, i.e. distribution in face vs. hand, in lips vs. upper face, and in left vs. right side of each AOI. For each trial, we computed the proportion of the fixation time spent in the two AOIs. Those values were logit-transformed before being entered in ANOVAs with the relevant within-subject factors (lexicality, frequency, phonological mismatch and final word predictability), plus group as between-subject factor when considering CS users. Post-hoc comparisons were then conducted with Tukey’s test.

As the fixation cross preceding each video was displayed between the coders’ eyes, we systematically discarded the first fixation of each trial if it fell in the eyes AOI.

## Results

### Behavioral assessment of cued speech comprehension

The deaf and hearing CS users were presented with CS sentence videos and asked to write them down. Each sentence was presented twice, and two transcriptions were required. No difference was found between the first and the second attempt in any group, and the two responses were averaged (Suppl Fig. 1).

Deaf CS users responded very accurately: an average of 98.63 ± 1.92% of phonemes were transcribed correctly. The performance of hearing CS users was twice lower (48.11 ± 25.37%; Mann-Whitney U = 460, *P* < 0.001). Moreover, performance was more variable across subjects in the hearing than in the deaf group (Levene’s test for variance comparison: F = 38.75, *P* < 0.001). The superiority of deaf over hearing CS users prevailed separately for the 3 levels of sentence difficulty (easy: U = 445.5, *P* < 0.001; intermediate: U = 425, *p* < 0.001; difficult: U = 459, *P* < 0.001). Finally, pairwise comparisons showed that deaf users performed better for easy than for intermediate sentences (U = 503, *P* < 0.001), and for intermediate than for difficult sentences (U = 47, *P* < 0.001). In hearing users, performance level did not differ with sentences difficulty (all *P* > 0.05).

### General gaze pattern between face and hand

A qualitative examination of the overall gaze distribution showed that, in the three groups and the two experiments, the gaze was almost exclusively directed towards the face, with foci around the lips and in the nose/eyes vicinity, and only marginally towards the hand (Fig. 1B). One participant from the deaf group showed a strikingly divergent behavior, mostly looking at the hand of the coder, and much less at the face. This pattern was different from all the other participants’ and present in all conditions of both experiments. This participant was excluded from further analyses. Because the other participants’ gaze was stable and directed at a small region, we only analyzed fixations. We will start by studying the pattern of fixation in CS users.

### Fixation pattern in cued speech users

Statistical analyses confirmed the massive predominance of the face over the hand with real words (97.2% face fixation), with pseudowords (96.7%), and with sentences (98.8%) (all *P* < 0.001).

The face area was further divided into two areas of interest (AOIs): one covering the lips and the other covering the upper face. About 6% of face fixation time was spent outside of those AOIs, spread in more peripheral regions of the face (real words: 5.5%; pseudowords: 5.8%; sentences: 7.2%). Using the same ANOVA model as before with the lips and upper face AOIs, we addressed the following questions, each time looking for group differences: First, disregarding finer-grained experimental factors, how was fixation shared between these two parts of the face, and between their left and right halves? Second, what was the influence on those parameters of lexicality, word frequency, word mismatch, and semantic predictability?

#### Gaze distribution between the lips and the upper face

**Fig. 2:**
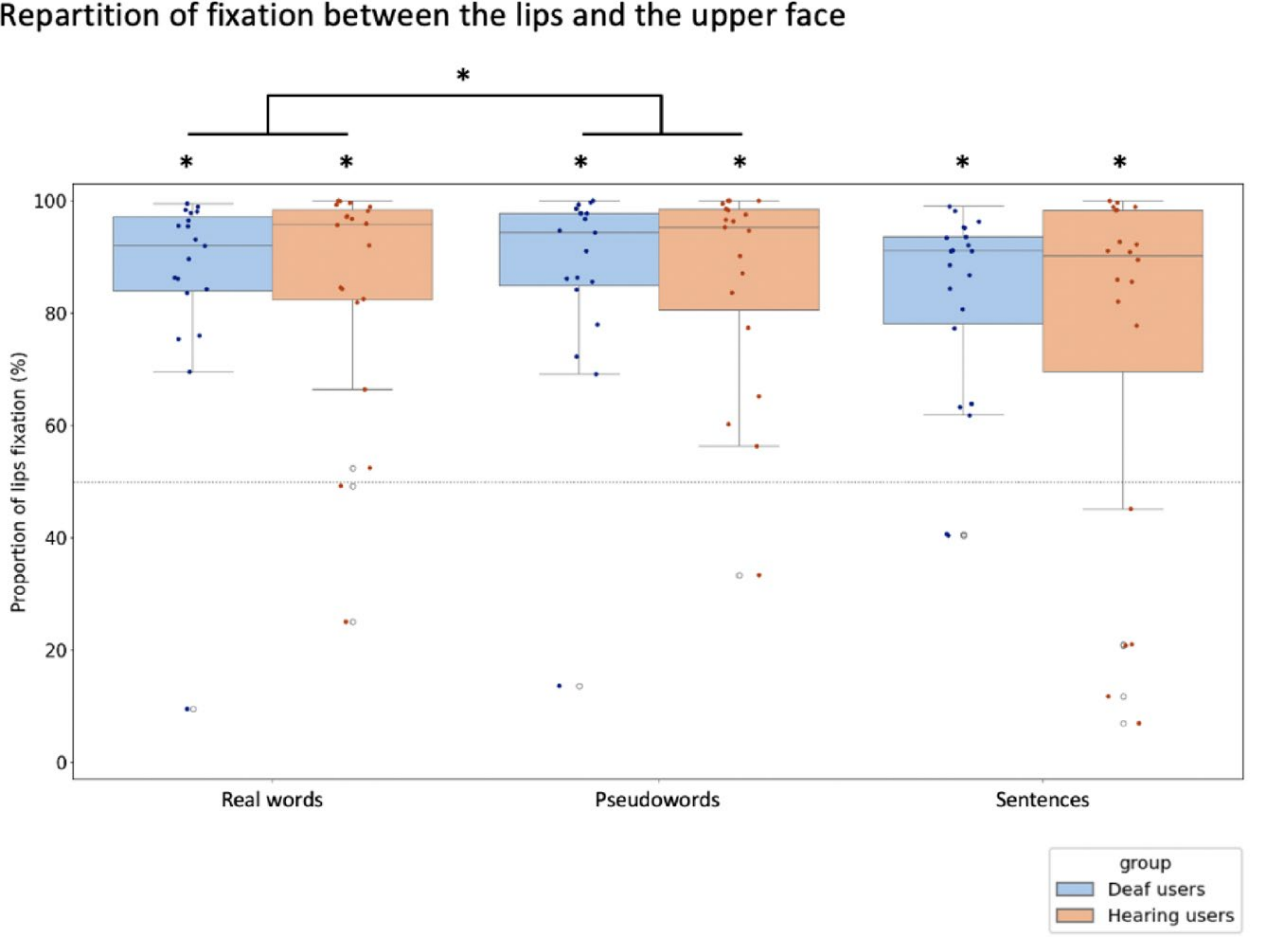
Proportion of fixation time spent on the lips during the perception of real words, pseudowords and sentences, in deaf and hearing users of cued speech. Significance: * *P* < 0.05; ** *P* < 0.01; *** *P* < 0.001.

As suggested by the inspection of gaze heat maps, most fixations were directed towards the lips (real words: lips 84.3%: pseudowords: 85.3%; sentences: 79.1%; *P* < 0.05; Fig. 2). This distribution did not differ between the deaf and hearing users.

#### Asymmetry of fixation

The whole face (including peripheral regions), and the upper-face and lips AOIs were each split in their left and right halves, and we studied fixation asymmetry separately for each AOI.

##### Whole-face analysis

We first examined the asymmetry of fixation to the face as a whole (Suppl Fig. 2). For words, there was a group difference (F(1,36) = 4.12, *P* < 0.05), with deaf CS users showing no asymmetry (left 47.9%) and hearing CS users showing a leftward bias (left 68.2%, *P* < 0.05).

For pseudowords, there was no difference between groups, with a significant left side predominance (left 62.3%; *P* < 0.05).

Sentences showed the same pattern as words, with no asymmetry in the deaf group (left 45.4%; *P* > 0.05), and a leftward bias in hearing participants (left 72.2%; *P* < 0.01; group difference F(1,40) = 9.13, *P* < 0.01).

We then assessed how this overall pattern reflected fixation asymmetry in the upper and in the lower parts of the face.

##### Fixation on the lips

**Fig. 3:**
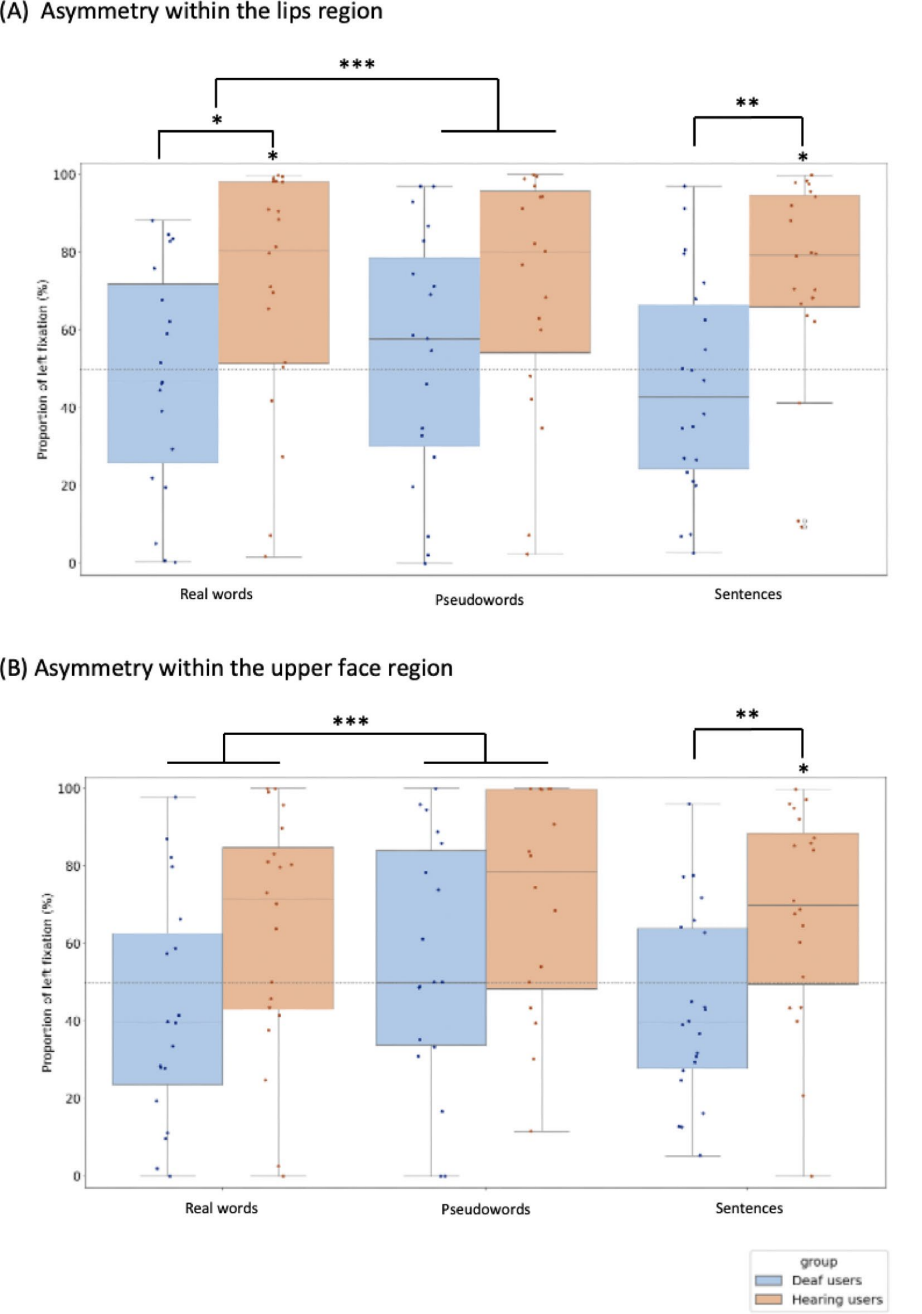
Proportion of fixation time spent in the left half of facial areas of interest, during the perception of real words, pseudowords and sentences, in deaf and hearing users of cued speech. (**A**) Within the lips region; (**B**) Within the upper face region. Significance: * *P* < 0.05; ** *P* < 0.01; *** *P* < 0.001.

The asymmetry on the lips region was similar to the whole-face pattern (Fig. 3A). With words, there was a significant group difference (F(1,36) = 4.36, *P* < 0.05): deaf users did not show a fixation asymmetry (left 48.7%), while hearing users had a leftward bias (left 71.5%, *P* < 0.05). With pseudowords, fixation did not differ between groups, and showed no asymmetry.

Sentences showed the same pattern as words (deaf users: left 45.3%, *P* > 0.05; hearing users: left 73.2%, *P* < 0.05; group difference F(1,40) = 11.38, *P* < 0.01).

##### Fixation on the upper face

For words, fixation did not differ between groups, and showed no asymmetry (Fig. 3B). Still, hearing participants showed a tendency for a leftward bias (left 58.2%) and deaf participants a tendency for a rightward bias (left 41.6%).

For pseudowords, fixation did not differ between groups, and showed no asymmetry, despite a tendency to fixate more on the left in both groups (deaf: left 57.5%; hearing: left 68.1%).

For sentences, deaf CS users showed no asymmetry (left 41.7%; *P* > 0.05), while hearing CS users fixated more on the left (left 72.1%, *P* < 0.05; group difference F(1,39) = 10.38, *P* < 0.01).

To summarize, in the lips region, there was a general leftward fixation bias in hearing participants, and no consistent asymmetry in the deaf group. Fixation to the upper face was roughly symmetrical in both groups, except for sentences, which followed the pattern of the lips region. We then studied whether lexical and semantic factors modulated the upper-lower and the left-right pattern of fixation.

#### Comparison of real words and pseudowords

We found no interaction involving the group factor.

##### Gaze distribution between the lips and the upper face

There was a significant effect of lexicality (F(1,36) = 6.06, *P* < 0.05). The usual predominance of fixation on the lips relative to the upper face was smaller with real words (lips 84.3%) than with pseudowords (lips 85.3%).

##### Asymmetry of fixation

At the whole-face level, there was a significant effect of lexicality (F(1,36) = 26.10, *P* < 0.001). There was a non-significant leftward tendency for real words (left 58.3%), which was significant for pseudowords (left 62.3%, *P* < 0.01).

We found the same pattern in the lips region (lexicality F(1,36) = 17.91, *P* < 0.001), with a non-significant leftward tendency for real words (left 60.1%, *P* > 0.05), and a significant left preference with pseudowords (left 63.7%, *P* < 0.05).

In the upper face, we found a main effect of lexicality (F(1,21) = 28.45, *P* < 0.001), with a stronger leftward bias in pseudowords (left 62.6%) compared to real words (left 49.5%).

#### Influence of frequency and syllabic-CS mismatch on the fixation of real words

We then examined the influence of real word frequency and syllabic-CS mismatch on the fixation pattern (Suppl Fig. 3). There was no interaction involving the group factor.

##### Fixation distribution between the lips and the upper face

There was no frequency x mismatch interaction. There was an effect of frequency (F(1,36) = 19.27, *P* < 0.001), with a stronger predominance of the lips for low- (lips 85.0%) than high-frequency words (lips 83.5%). There was also an effect of mismatch (F(2,72) = 7.49, *P* < 0.01), with a stronger predominance of the lips when the word had level-2 mismatch than when it had no or level-1 mismatch (fixation on the lips for mismatch levels 0: lips 84.1%; 1: 84.1%; 2: 84.6%; post-hoc comparisons between mismatch levels 0–1: t(36) = -1.02, *P* > 0.05; 0–2: t(36) = -3.05, *P* < 0.05; 1–2: t(36) = -3.16, *P* < 0.01).

##### Asymmetry of fixation

We then examined the left-right asymmetry of fixation inside each AOI separately. The analyses showed no interaction involving the group factor.

At the whole face level, we found a main effect of frequency (F(1,36) = 4.15, *P* < 0.05), with a larger leftward bias with frequent (left 58.9%) than with infrequent words (left 57.6%). There was a frequency x mismatch interaction (F(2,72) = 19.95, *P* < 0.01). High-frequency words showed a larger leftward bias with mismatching than with matching words (no mismatch: left 57.6%; level-1: 58.5%; level-2: 58.7%; comparisons between mismatch levels 0–1: t(36) = -3.77, *P* < 0.01; 0–2: t(36) = -5.03, *P* < 0.001; 1–2: t(36) = 0.26, *P* > 0.05). Low-frequency words showed no difference between mismatch levels (all post-hoc pairwise comparisons *P* > 0.05).

In the lips region, we found a frequency x mismatch interaction (F(2,72) = 10.94, *P* < 0.001), with the same pattern as for the whole face. With high-frequency words, high mismatch attracted the gaze to the left more than low mismatch (mismatch level 0: 56.9%; 1: 61.9%, 2: 61.3%; comparisons between mismatch levels 0–1: t(36) = -3.80, *P* < 0.01; 0–2: t(36) = -3.38, *P* < 0.05, P; 1–2: t(36) = 0.06, *P* > 0.05). There was no effect of mismatch with low-frequency words (mismatch level 0: 60.5%; 1: 59.0%, 2: 59.4%; all *P* > 0.05). The effect of frequency was not significant, either as a main factor or in post-hoc tests restricted to the 3 levels of mismatch.

In the upper face, we found a main effect of frequency (F(1,32) = 20.28, *P* < 0.001), with a stronger leftward tendency with high-frequency words (left 54.1%) compared to low-frequency words (left 44.5%; post-hoc t(36) = -4.03, *P* < 0.001).

#### Influence of predictability on the fixation of last words of sentences

We analyzed the fixation pattern during the last word of predictive and non-predictive sentences, starting at the beginning of the coder’s production of the last word. There was a significant effect of predictability at the whole face level (F(1,40) = 12.53, *P* < 0.01), with no difference between groups, with a larger leftward bias when the final word was unpredictable (left 57.9%, *P* > 0.05) than when it was predictable (left 56.2%, *P* > 0.05).

### Fixation pattern in hearing controls

**Fig. 4:**
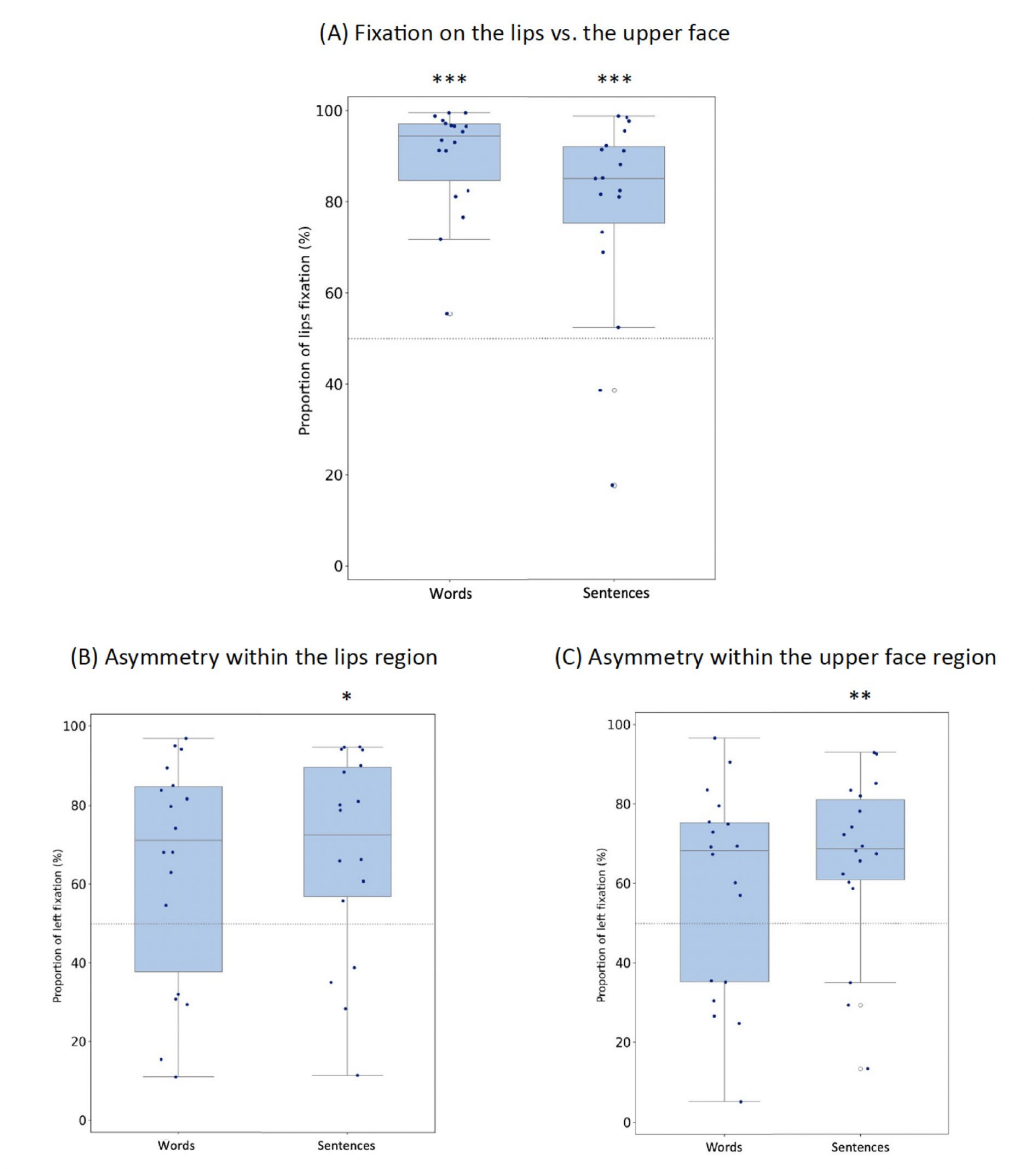
Gaze pattern in controls. (**A**) Lips-upper face repartition of fixation; Asymmetry of fixation (**B**) Within the lips region (**C**) Within the upper face region. Significance: * *P* < 0.05; ** *P* < 0.01; *** *P* < 0.001.

We then analyzed fixation in control participants (Fig. 4). As these participants are ignorant of cued speech, they did not perform the same task, and received only a small subset of stimuli in a short experiment. We therefore assessed only the main parameters of fixation.

#### Fixation pattern across areas of interest

As for CS users, hearing controls spent most of the time looking at the coder’s face, and almost never fixated the hand (real words: 96.4% face fixation; pseudowords 96.6%; sentences: 96.6%; all *P* < 0.001).

About 6% of fixation time was spent in the periphery of the face (real words: 5.5%; pseudowords: 5.8%; sentences: 5.5%). Fixation was again mostly directed towards the lips relative to the upper face (real words: lips 89.6%; pseudowords: 91.5%; sentences: 78.8%; all *P* < 0.001; Fig. 4A).

#### Asymmetry of fixation

We then examined the left-right asymmetry of eye gaze among the samples directed at the head in control participants (Fig. 4B). Control participants presented a leftward bias at the whole face level, significant when considering pseudowords and sentences (real words: left 63.6%, *P* > 0.05; pseudowords: 65.6%, *P* < 0.05; sentences: 67.2%, *P* < 0.05). The same pattern was observed at the lips level (real words: left 63.8%, *P* > 0.05; pseudowords: 65.5%; *P* < 0.05; sentences: 67.8%, *P* < 0.05).

Considering the upper face level, fixation presented no asymmetry for real words (left 49.4%) nor for pseudowords (left 53.5%). There was a leftward bias for sentences (left 66.6%, *P* < 0.01).

## Discussion

We found that all groups fixated almost exclusively on the face, and predominantly on the lips of the speaker. Deaf and hearing participants differed strikingly in the way fixation was distributed between the left and right halves of the face. While both hearing groups mostly fixated the left side of the speaker’s face, deaf participants showed a more symmetrical pattern. Finally, in CS users, stimuli that were phonologically, lexically or semantically more difficult tended to increase fixation in the inferior and left sector of the face.

We will now discuss in turn the predictions and questions formulated in the introduction.

### Gaze distribution between the hand and the face, and between the eyes and the mouth

The fixation pattern which we observed during CS perception by CS users was, in its main features, similar to the behavior of hearing persons watching and hearing a speaking face, with or without accompanying expressive hand gestures. As reviewed in the introduction, this pattern consists of almost exclusive fixation of the face, and predominant fixation of the mouth region as compared to the eyes^18–20^. Deaf and hearing users of CS did not differ, and showed the same overall behavior as naïve controls. This observation, which validates our first two predictions, confirms that this default gaze strategy may be optimal for visually gathering phonological information from a speaking face, information which is virtually the same during audiovisual and CS perception. Moreover, like for gestures ancillary to audiovisual speech, for signed languages, and for fingerspelling, CS gestures are exploited without requiring direct fixation to the hand. This is true even though several evidence suggest that CS gives some conceptual primacy to gestures^2,6,8,9^, and even though gestural cues occur slightly earlier than mouth-related cues^4,5,10^. Still, the absence of group differences despite the marked superiority of the deaf group at lip-reading may come as a surprise, assuming that their superiority reduced the need for intensive fixation of the mouth. One may speculate that the non-interactive videos which we presented induced little social motivation to look at the coder’s eyes and away from the mouth^34,37,82^.

### Gaze distribution between the left and the right sides of the face

The left-right distribution of fixation is the one parameter on which hearing and deaf users of CS differed. On the one hand, hearing CS users showed a predominance of fixation to the left in the mouth region, but also to some extent in the upper face for the more ecological full sentences, in agreement with our third prediction. The control participants showed the same overall pattern. As argued before about the eyes-mouth bias, this leftward bias is the default behavior also observed whenever hearing individuals are watching and hearing a speaking face, likely driven by the left lateralization of the most informative mouth movements^66,67^. In the present study this behavior may be amplified by the presence of CS gestures on the left side, which should act both as low-level attractors and as relevant information for CS users.

In contrast, the deaf participants showed a symmetrical gaze distribution for all types of stimuli in the critical mouth region. In the upper face, deaf participants were again asymmetrical in the only situation in which the hearing group showed a leftward bias, that is with sentences. The presence of hand gestures at the left of the speaker’s face and arguably processed by deaf CS users was not sufficient to bias left-right fixation asymmetry in this group. This finding should be discussed in the context of other peculiar patterns of asymmetry of visual perception in deaf people. Thus, in studies targeting non-verbal facial features such as emotion or gender, deaf people show a reduced or absent left visual field advantage for faces presented in one hemifield^83–85^, and even in central position^86^. More relevant to the current issue, the usual right visual field advantage for processing written words is reduced or absent in deaf persons, for instance in tasks of word identification^69^ or rhyme judgment^87^. There is also an expanded visual reading span to the left of fixation, from 4 characters in hearing controls to 10 characters in deaf individuals^70^. Such converging observations suggest that a general increase in the ability to process linguistic information from the left hemifield may explain why deaf participants fixate faces symmetrically and yet perceive hand gestures efficiently and are experts at lip-reading. Moreover, there is enhanced processing of visual stimuli in the peripheral visual field of deaf compared to hearing people, although the literature is not fully consistent on this matter, possibly due to the heterogeneity of the deaf population^71,72^. This phenomenon could contribute to erasing the usual leftward fixation bias, by making gestures easy to perceive despite their distance to the left of the fovea. Although the exact roles of deafness and CS mastery may be difficult to disentangle, the current data suggest that hearing the sounds of language may be important to develop the typical cerebral left-lateralization for the visual processing of phonological cues.

### The impact of stimulus difficulty

Finally, we compared stimuli along various language-related dimensions of lexicality, frequency, phonological mismatch, and semantic predictability. We predicted that the effect of those variables could be at least partly reduced to the impact of processing difficulty, assuming that higher difficulty would require a more active and longer research of information, and hence more time spent in the most informative regions. The results support this overall interpretation. First, pseudowords reproducibly induce more errors and slower response latencies than real words during reading^88^, audio and audiovisual speech perception^89^, and CS perception^1^. We showed that they also yielded longer fixation to the mouth and to the left side of the face, which carry most facial phonological information^64,65^. Second, infrequent words are more difficult to identify than frequent ones visually and auditorily^90^, and frequency modulates eye fixation during reading^91^. Compatible with a general interpretation in terms of difficulty, we found that a lower word frequency also draws the gaze down to the mouth during CS perception. Third, phonological mismatch had a similar impact. Spoken words which include consonant clusters or CVC syllables need to be re-parsed to generate their CS form, resulting in more numerous CS gestures than spoken syllables. Such words are more error prone^1^, and we showed accordingly that mismatch increases the time spent fixating the mouth. Moreover, it draws fixation towards the left half of the mouth, as predicted, but only for high-frequency words. This interaction of mismatch with frequency may reflect a ceiling effect. Fixation to the left of the mouth is already at a ceiling level for low-frequency words, irrespective of their degree of mismatch. For the easier high-frequency words, the difficulty incurred by mismatch increased fixation to the left side of the mouth. Finally, we compared fixation to words following a predictive or an non-predictive context. Predictability facilitates the recognition of spoken and written words^92,93^ and has an impact on eye movements during reading^94^. We found that during CS perception, the fixation of non-predictable words, which require better information capture, is biased to the left.

In summary, the impact of our manipulation of phonological, lexical, and semantic parameters may be explained on the basis of comprehension difficulty, more difficult stimuli attracting fixation towards the left half of the mouth region, in both deaf and hearing users of cued speech. It is noteworthy that hearing CS users were sensitive to difficulty, although their comprehension of CS was moderate (Supplementary Fig. 1). Two mechanisms may explain these results: (i) the comprehension capacities of hearing users may be sufficient to evaluate difficulty and to adjust their behavior accordingly, (ii) difficulty may influence CS production parameters (e.g. production speed, gesture amplitude), so that participants may be sensitive to these low-level variations in the stimuli.

## Conclusion

By supplementing spoken language with hand gestures, cued speech stands as the sole system for visually conveying full phonological information of a spoken language, apart from reading. We found that the overall pattern of fixation was close to the default gaze behavior prevailing also during audiovisual speech perception, with substantial deviation from this pattern mostly occurring in deaf participants. Thus, all groups fixated almost exclusively on the face, and predominantly on the lips of the speaker, despite the effective processing of CS gestures by CS users. Deaf and hearing participants differed strikingly in the way fixation was distributed between the left and right halves of the face. While both hearing groups mostly fixated the left side of the speaker’s face, deaf participants showed a more symmetrical pattern. Finally, in CS users, stimuli that were phonologically, lexically or semantically more difficult tended to increase fixation in the inferior and left sector of the face. This study thus elucidates the fundamental behavioral tuning that facilitates the efficient recovery of phonology in this distinctive form.

## Supplementary Information

Below is the link to the electronic supplementary material.

Supplementary Material 1

## Data Availability

Eye-tracking data, behavioral data and experimental material supporting the findings of this study will be made available from the corresponding author, upon reasonable request. The stimulation and analysis scripts are available at https://github.com/AnnahitaSarre/CUSTIME_gaze_dynamics.
