# Supplementary material for: Gaze dynamics of cued speech perception
Source: Sci Rep. 2026 Jun 12;16:18210. doi: 10.1038/s41598-026-40719-9 (PMC13260914; doi:10.1038/s41598-026-40719-9)
Supplement: Supplementary file 1 — Supplementary Material 1 [file 41598_2026_40719_MOESM1_ESM.pdf]

Supplementary figure 1

CS comprehension ability in CS users

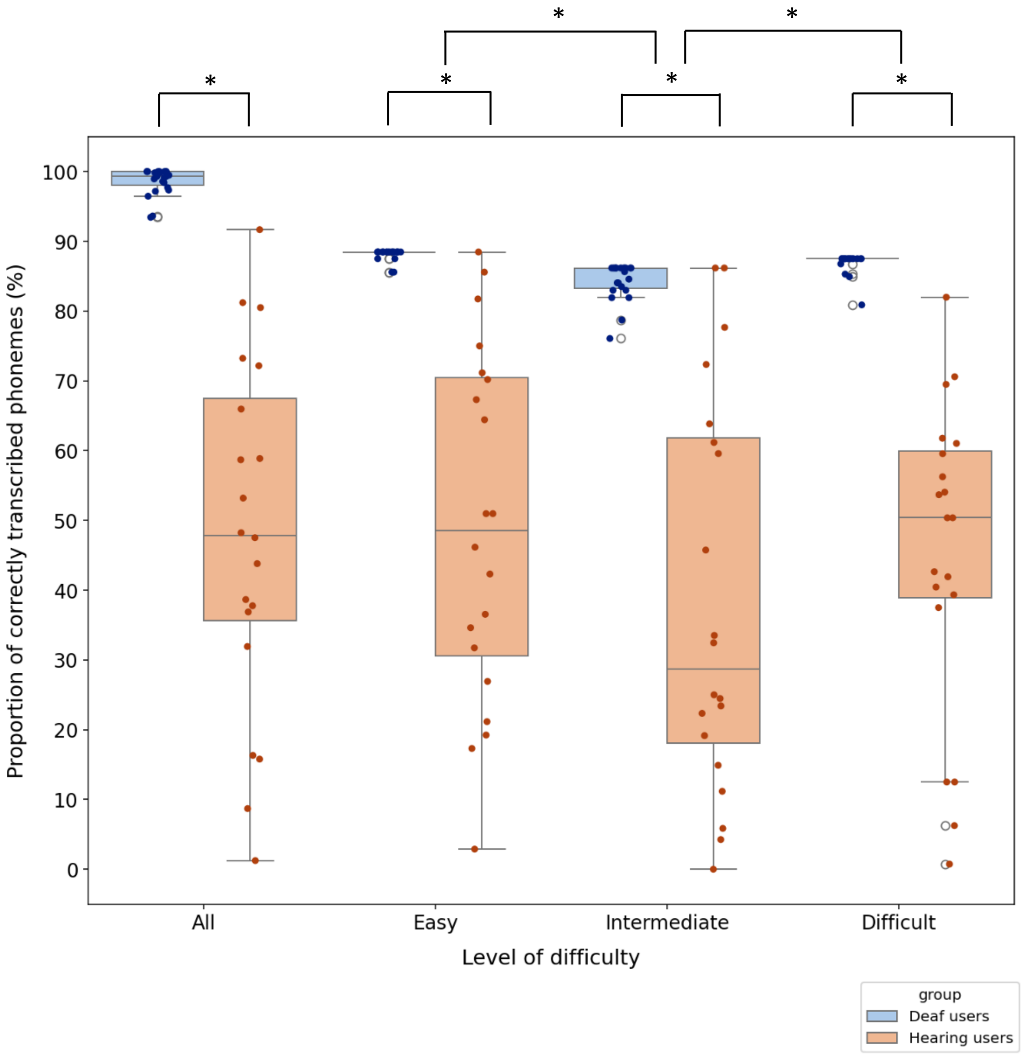

Supplementary figure 1. CS comprehension accuracy, as indexed by the percentage of correctly transcribed phonemes, in deaf and hearing CS users, for all sentences and for each level of difficulty. Hearing CS users performed less well, and with larger individual variability, than deaf participants. Significance: * P<0.05; ** P<0.01; *** P<0.001

Supplementary figure 2

Asymmetry within the face region

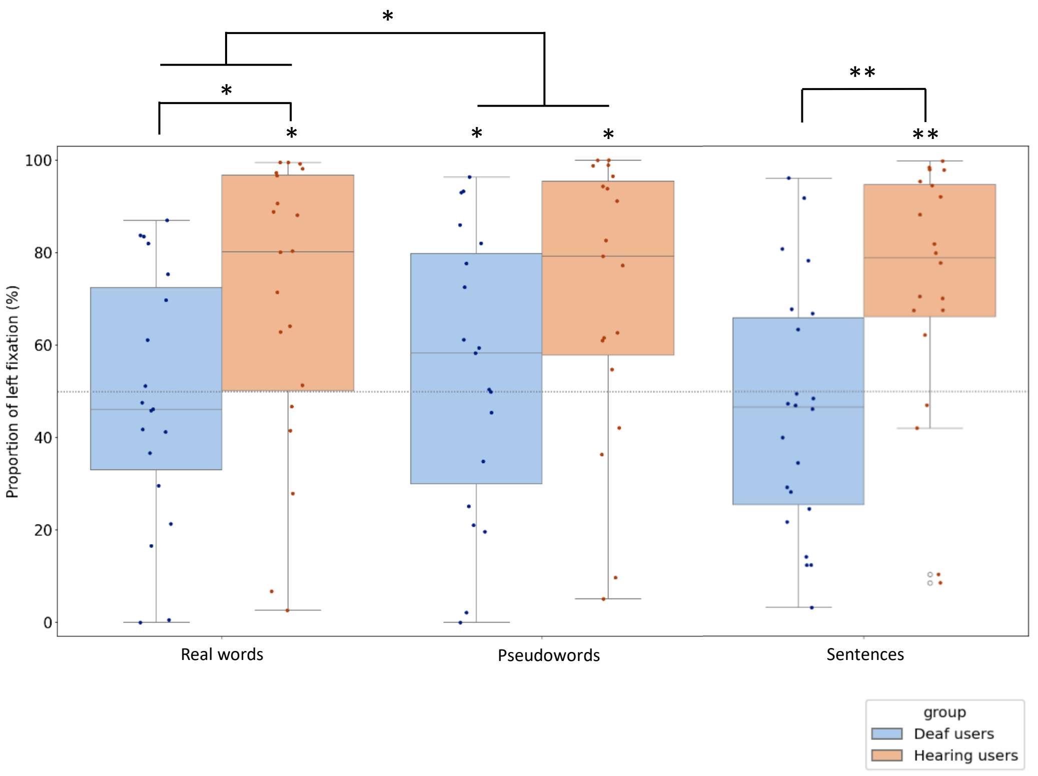

Supplementary figure 2. Left-right asymmetry of fixations in CS users within the whole face. Significance: * P<0.05; ** P<0.01; *** P<0.001

(A) Asymmetry within the lips region

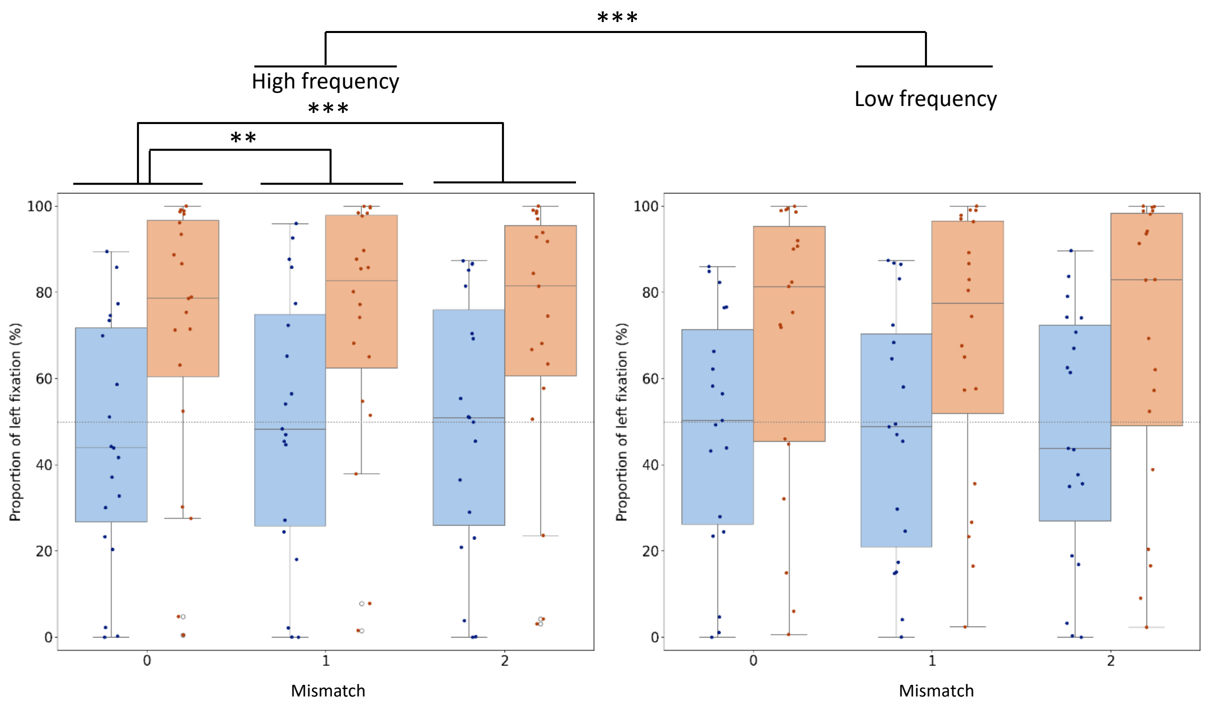

(B) Asymmetry within the upper face region

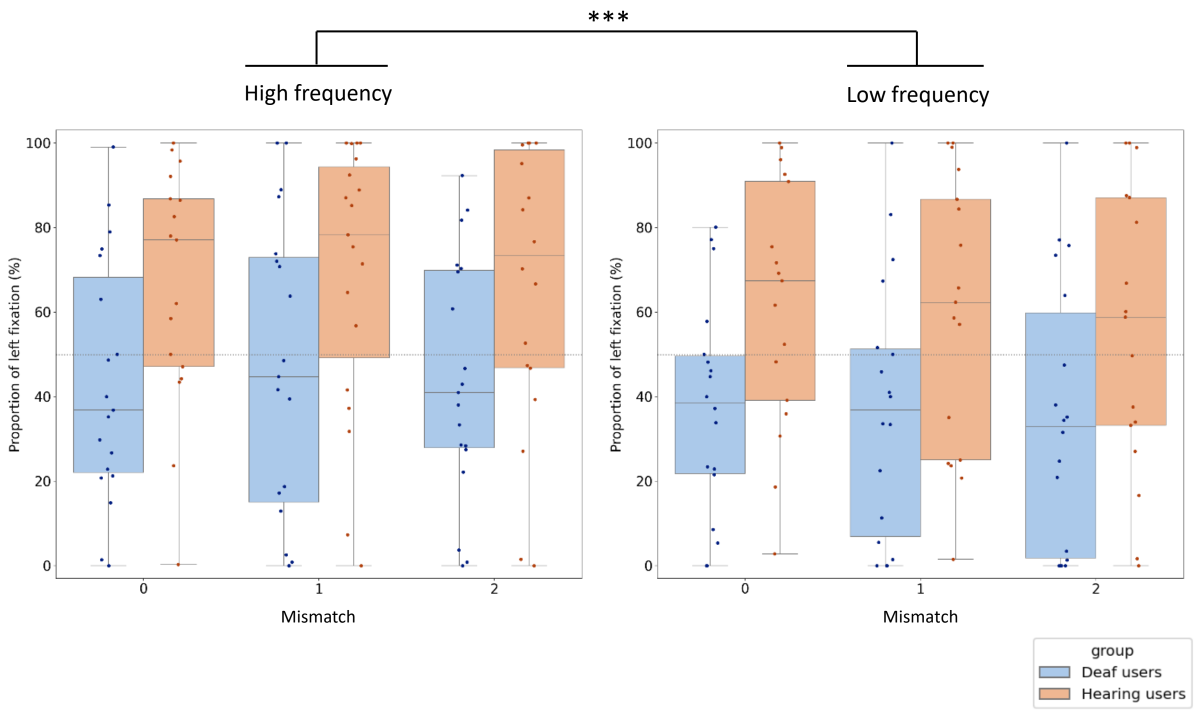

Supplementary figure 3. Influence of word frequency and level of mismatch on the left-right asymmetry of fixation in CS users. (A) Within the lips region. (B) Within the upper face region. Significance: * P<0.05; ** P<0.01; *** P<0.001
